# Research on Visual–Inertial Measurement Unit Fusion Simultaneous Localization and Mapping Algorithm for Complex Terrain in Open-Pit Mines

**DOI:** 10.3390/s24227360

**Published:** 2024-11-18

**Authors:** Yuanbin Xiao, Wubin Xu, Bing Li, Hanwen Zhang, Bo Xu, Weixin Zhou

**Affiliations:** 1 School of Mechanical and Automotive Engineering, Guangxi University of Science and Technology, Liuzhou 545006, China; xyb15766249296@163.com (Y.X.); zhw14780050109@163.com (H.Z.); xu773195587@163.com (B.X.); 18779767166@163.com (W.Z.); 2 Guangxi Collaborative Innovation Centre for Earthmoving Machinery, Guangxi University of Science and Technology, Liuzhou 545006, China; 3 Guangxi Electrical Polytechnic Institute, Nanning 530007, China

**Keywords:** open-pit mine, SLAM, point feature fusion, line feature extraction

## Abstract

As mining technology advances, intelligent robots in open-pit mining require precise localization and digital maps. Nonetheless, significant pitch variations, uneven highways, and rocky surfaces with minimal texture present substantial challenges to the precision of feature extraction and positioning in traditional visual SLAM systems, owing to the intricate terrain features of open-pit mines. This study proposes an improved SLAM technique that integrates visual and Inertial Measurement Unit (IMU) data to address these challenges. The method incorporates a point–line feature fusion matching strategy to enhance the quality and stability of line feature extraction. It integrates an enhanced Line Segment Detection (LSD) algorithm with short segment culling and approximate line merging techniques. The combination of IMU pre-integration and visual feature restrictions is executed inside a tightly coupled visual–inertial framework utilizing a sliding window approach for back-end optimization, enhancing system robustness and precision. Experimental results demonstrate that the suggested method improves RMSE accuracy by 36.62% and 26.88% on the MH and VR sequences of the EuRoC dataset, respectively, compared to ORB-SLAM3. The improved SLAM system significantly reduces trajectory drift in the simulated open-pit mining tests, improving localization accuracy by 40.62% and 61.32%. The results indicate that the proposed method demonstrates significance.

## 1. Introduction

Traditional mining operations are gradually moving toward automation and intelligence with the development and deployment of intelligent mining technology. Mining production safety and efficiency have increased dramatically, relying on unmanned operation, low-carbon processes, and intelligent equipment [1]. Nonetheless, the intricate topography, rough surface, and fluctuating illumination in open-pit mines present significant challenges to the current SLAM (Simultaneous Localization and Mapping) systems. Traditional methods, such as RFID and ZigBee, fail to deliver accurate localization in such environments, making visual SLAM a viable yet imperfect alternative due to its susceptibility to feature extraction failures under dynamic scenarios, low-lighting conditions, and complex terrain [2]. Therefore, an enhanced SLAM algorithm that integrates multi-sensor data, particularly IMU data, is essential for overcoming these issues in open-pit mining. This paper aims to address these limitations by developing a novel approach specifically designed for rugged mining environments, where traditional SLAM techniques often encounter significant challenges.

SLAM systems rely on multi-modal sensors attached to mobile robots for real-time perception and digital map construction of intricate environments with high accuracy, using sensor fusion methodologies. In mining applications, previous work [3] introduced a visual–inertial–wheel odometry method, offering robust initialization and accurate estimates for ground robotics through wheel encoder data, helping resolve initialization scale issues in straight-line motion. However, our study advances this approach by directly addressing the challenges of feature extraction accuracy and robustness in open-pit mining, incorporating more effective data fusion techniques for improved localization. Ref. [4] enhanced ORB feature and line feature matching algorithms for low-light, intricate terrain in coal mine tunnels, increasing tracking stability and accuracy despite monocular depth limitations. Similarly, methods such as CI-TEDM have been developed to enhance RGB-D SLAM accuracy in weakly textured scenes by incorporating wheel encoder data for stability [5]. Despite these advancements, most existing studies focus on controlled or indoor environments and do not sufficiently address the challenges posed by open-pit mining, which our work specifically targets. Furthermore, point cloud registration techniques have been proposed to enhance registration accuracy by mitigating feature degradation issues due to local sparsity and motion distortion in coal mine tunnels [6]. SLAM approaches that integrate depth information with visual features have also been developed to improve trajectory accuracy in complex scenes, combining tracking, local mapping, and loop detection [7].

In recent years, many researchers have actively explored methods to improve SLAM performance in complex environments. For example, comprehensive reviews on obstacle avoidance and path planning have highlighted the importance of integrating global path planning with local obstacle avoidance for efficient navigation in complex and dynamic environments [8]. This integration is crucial for environments like open-pit mines, where obstacles and terrain irregularities are unpredictable. Ye et al. [9] developed the IKB-RRT algorithm, optimizing path smoothness in complex orchard environments. In contrast, our study emphasizes continuous localization accuracy and map reliability in open-pit mines. Hybrid approaches that combine knowledge-driven and data-driven methodologies have also been proposed to enhance system stability under variable conditions [10]. These strategies offer valuable insights for improving SLAM robustness in unpredictable mining environments. In addition, monocular frame optimization techniques based on parallax analysis have been applied in constrained spaces, providing valuable insights for SLAM applications in environments with limited geometric cues [11]. Other studies have contributed to line segment extraction and feature matching techniques to enhance SLAM capability in complex scenarios. For instance, methods for extracting line segments from large-scale point clouds using weighted centroid displacement have improved geometric edge detection, providing technical support for SLAM systems in complex scenarios [12]. Additionally, line feature detection methods based on the Spherical Hough Transform have improved point cloud and image matching accuracy, emphasizing the role of multi-sensor fusion in enhancing SLAM robustness [13]. These approaches have been instrumental in advancing SLAM performance in dynamic and unstructured environments, although challenges remain in adapting these methods to open-pit mining conditions.

Building upon these foundations, we aim to enhance localization accuracy and robustness through effective data integration and feature extraction improvements. For example, prior approaches like VINS-MONO have shown how visual and IMU data integration can improve localization precision [14]. Yet issues such as lighting variability continue to impact performance in certain conditions, necessitating further advancements. Similarly, feature extraction techniques in ORB-SLAM systems [15,16] and direct methods like DSO [17] demonstrate the importance of feature quality and tracking accuracy but are often constrained by hardware limitations in low-texture environments. PL-VIO, which incorporates line features into VINS-MONO, exemplifies efforts to combine multiple features for robust tracking, though it incurs high computational costs [18]. Our approach expands on these methods by directly addressing the specific requirements of open-pit mining, where factors like low-texture terrain and viewpoint jitter present significant challenges.

This paper contributes a unique SLAM localization approach specifically adapted for open-pit mines by employing an improved LSD line segment detector, which utilizes short segment culling and approximate line merging strategies to enhance feature extraction quality. Through the close integration of point and line features with IMU data and a sliding window optimization technique, our method achieves higher accuracy in pose estimation and resilience against feature loss due to viewpoint jitter in low-texture settings. By addressing the weaknesses of existing SLAM systems, this research provides a more reliable and accurate SLAM framework for complex and unpredictable mining environments, achieving high-precision localization and environmental mapping under extreme conditions.

## 2. Visual–Inertial Fusion SLAM System Framework

**Figure 1:**
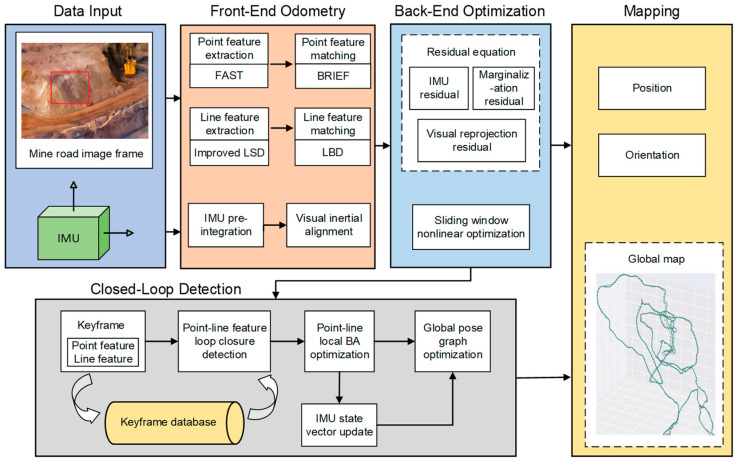
The system framework diagram. This procedure encompasses data input, front-end visual–inertial odometry, closed-loop detection, back-end optimization, and mapping; The red box in the data input section represents the sparse textured slope.

This work presents a SLAM system that employs nonlinear optimization for posture estimation by combining point and line visual characteristics with IMU data. This procedure encompasses data input, front-end visual–inertial odometry, closed-loop detection, back-end optimization, and mapping. The comprehensive system framework is illustrated in Figure 1.

### 2.1. Front-End Visual–Inertial Odometry

The front-end visual odometry module preprocesses data obtained from the camera and IMU. This procedure entails concurrently processing raw pictures to identify and monitor point and line features. The extraction of point features employs the oFAST (FAST Keypoint Orientation) technique from ORB-SLAM3, which identifies keypoints by detecting intensity variations between pixels and their adjacent counterparts. It also computes the feature points’ principal orientation to improve the BRIEF descriptor’s rotational invariance. Line feature extraction utilizes an enhanced LSD (Line Segment Detection) algorithm, integrating segmentation filtering and adjacent line merging techniques to extract essential line features while efficiently eliminating superfluous line segments. In feature matching, the Line Band Descriptor (LBD) [19] monitors and matches the extracted line segments. At the same time, geometric constraint methods are applied to minimize mismatches, ensuring matching accuracy. Furthermore, during system initialization, IMU data from the gyroscope and accelerometer are pre-integrated, and the pose and velocity of the current frame are inferred based on the pose variations between consecutive visual frames [20]. The system achieves unified initialization by concurrently processing optical and inertial data in the front-end odometry, enhancing both accuracy and stability.

### 2.2. Back-End Nonlinear Optimization

During the back-end optimization phase, the system performs nonlinear optimization on the pose, formulating an objective function encompassing visual reprojection residuals, IMU pre-integration residuals [21], and marginalization residuals. The objective function is addressed by a nonlinear least squares technique, incorporating limitations from various sensor observations. To balance accuracy with computational efficiency, the back-end optimization employs a sliding window technique that concurrently minimizes the residual function to ascertain the best state vector, encompassing pose, velocity, spatial points, spatial lines, and the accelerometer and gyroscope biases. To maintain system correctness and real-time performance during prolonged operation, when a new image frame is introduced, the most recent frame in the sliding window is processed via a marginalization model, preserving the window size and assuring efficient system functionality.

### 2.3. Loop Closure Detection

The loop closure optimization thread utilizes a visual loop closure detection technique grounded in the DBoW bag-of-words model, which identifies loop closures by evaluating the similarity of images. Upon detecting a probable loop closure, an initial spatiotemporal consistency assessment is conducted to validate the accuracy of the detection. Upon confirmation of loop closure, the system employs the Iterative Closest Points (ICP) [22] algorithm to accurately align the point clouds associated with the loop frames, producing precise loop closure constraints.

## 3. Front-End Visual–Inertial Odometry

### 3.1. Enhancement of Line Feature Detection Algorithm

**Figure 2:**
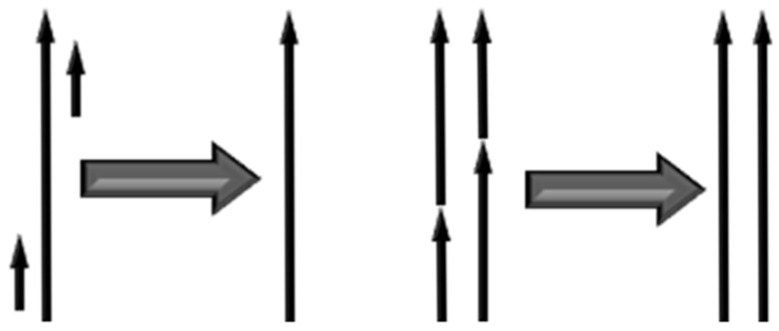
An example diagram of the line feature extraction optimization method. The efficacy of line segment identification is enhanced by implementing short line elimination and approximate line segment amalgamation procedures.

In the visual–inertial SLAM system that incorporates both point and line features, line features offer supplementary geometric restrictions for the pose estimate of the mobile device. Consequently, the line feature detection technique requires extracting salient line features from the image, eschewing the dependence on extra line features to develop a comprehensive environmental model. The LSD method identifies significant pixel clusters utilizing picture gradient data and row–column scanning techniques. Nevertheless, the absence of efficient procedures for merging and filtering line segments results in numerous discovered segments being redundant, analogous, or unable to satisfy the requisite length criteria. These subpar line segments not only elevate the computational demands in detection, description, and tracking but also considerably impair the performance and precision of the front-end visual–inertial odometry, thereby augmenting the uncertainty of the SLAM system during localization and mapping. This study proposes an enhanced LSD algorithm by parameter optimization, short segment elimination, and approximate line merging procedures to increase line segment identification quality. The enhanced LSD algorithm guarantees robust line feature extraction while minimizing computing time, as demonstrated in Figure 2.

**Figure 3:**
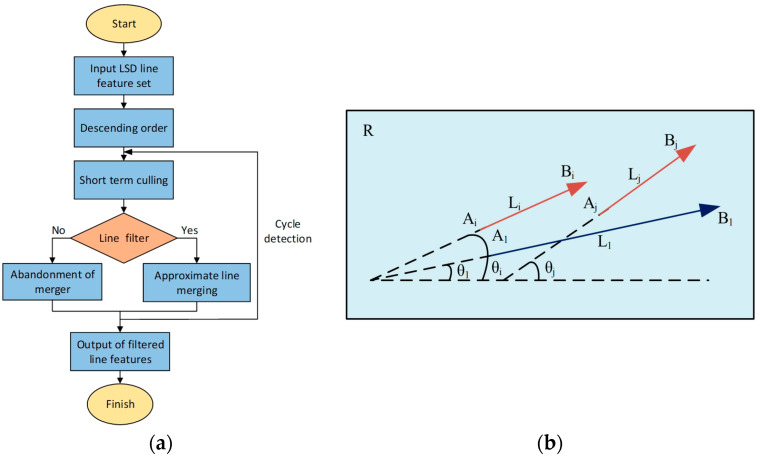
(**a**) Flowchart of improved LSD line feature detection algorithm; (**b**) schematic diagram of similar line feature merging.

Figure 3a illustrates the enhanced procedure of the LSD line feature identification algorithm, with the detailed implementation steps outlined below:

(1) Assume that the LSD algorithm detects all line features, denoted as L. Arrange these line features in descending order based on their length, $l_{i}$. The objective of sorting is to prioritize lengthy line segments, as they are more accurate and reliable in pose estimation and more stable in multi-image detection. The sequence of line segments following the arrangement is denoted as follows:

(1) $$L=\{l_{1},l_{2},l_{3},\cdots l_{m}\}$$

(2) Short segment elimination. To improve the reliability of the extracted line features, the algorithm eliminates segments that are too short to contribute meaningfully to pose estimation. Short line segments are often susceptible to noise and may not be stable across multiple frames. To address this, a minimum length threshold $len_{\min}$ is defined, ensuring only segments longer than this threshold are retained. The threshold is calculated as follows:

(2) $$len_{\min}=\left|\eta \cdot \min(W_{1},H_{1})\right|$$

where $len_{\min}$ represents the minimum length threshold, $H_{1},W_{1}$ denotes the height and width of the input image, $\left|*\right|$ is the rounded-up value, and η signifies the scale factor. In this study, η represents the scaling factor for the minimum length threshold. Based on experiments, η is set to 0.13 to effectively exclude shorter line segments while ensuring that longer segments enhance posture estimation accuracy and stability.

(3) Line Segment Selection. The selection process in sequence l commences with the most extended line segment $l_{1}$. Utilizing 2D Euclidean distance calculations for each image frame, which necessitates several screenings based on a distance threshold, will considerably elevate computational cost, compromising the algorithm’s real-time efficacy. This study employs a stepwise filtering method, wherein line segments are independently filtered based on vertical and horizontal distances, substituting the conventional 2D Euclidean distance computation. This approach, founded on basic absolute value operations, efficiently decreases computing expenses. The technique commences with horizontal distance filtering, which is succeeded by angle filtering for the line segment group that satisfies the horizontal distance criterion. Subsequently, vertical distance filtering is implemented. This stratified filtering approach substantially decreases the number of line segments processed per round, hence markedly enhancing filtering efficiency. The selection criterion for the candidate line segment group $l_{\alpha }$ that meets the horizontal distance filtering is as follows:

(3) $$\begin{array}{l} l_{\alpha }=\{\forall l_{m}\in l:(\left|\theta_{m}-\theta_{1}\right|<\mu \theta_{\min})\}\\ \mu =1-\frac{1}{1+e^{-4\lambda_{m}+5}},\lambda_{m}=\frac{k_{m}}{k_{1}} \end{array}$$

Among them, $l_{m}$ is a line segment in the line segment sequence, with a length of $k_{m}$ and an angle of $\theta_{m}$ in the horizontal direction. The angle screening threshold, $\theta_{\min}$, is used to assess the degree of approximation of the line feature angle. This paper establishes the value as π/90 following experimental analysis. μ is the adaptive proportional coefficient, inversely proportional to $k_{m}$. As $k_{m}$ decreases, the proportional coefficient μ increases, and the likelihood of the line segment merging increases. The mergeable line segments can be classified into two distinct end-to-end connection scenarios based on their position: head and tail, as illustrated in Figure 3b.

The merging operation is divided into two cases: head-end merging and tail-end merging, based on the varying positions of the mergeable line segments. The approximate line segment merging candidate group $l_{\beta }$ is derived by assuming that the line segment $l_{a},l_{b}$ is composed of two approximate line segments, with the head and tail endpoints being $\left(x_{a1},y_{a1}),(x_{a2},y_{a2}\right)$ and $\left(x_{b1},y_{b1}\right),\left(x_{b2},y_{b2}\right)$, respectively.

(4) $$l_{\beta }=\{\forall l_{a},l_{b}\in l_{\alpha }:\left(\left|x_{a1}-x_{1}\right|<f_{\min}\right)or\left(\left|y_{b1}-y_{1}\right|<f_{\min}\right)$$

where $f_{\min}$ represents the distance screening threshold for the vertical and horizontal distance approximation of the line segment. This study establishes the value of $f_{\min}$ as 3 following experimental investigation.

(4) Approximate merging of line segments. Based on the aforementioned screening criteria, the approximate line segment group $l_{\theta }=\{l_{\theta 1},l_{\theta 2}\}$ is established, followed by the addition of $l_{1}$ to the approximate line segment group $l_{\theta }$, resulting in a new line segment sequence $\left\{l_{1},l_{\theta }\right\}$. The initial and terminal endpoints that exhibit the most significant deviation are designated as the first and last endpoints of the new line segment $l_{r}$, and the angle $\theta_{r}$ of $l_{r}$ is recalculated. Upon the establishment of $\theta_{r}<\theta_{\min}$, the merger occurs, and line segment group $\left\{l_{1},l_{\theta }\right\}$ is supplanted by the new line segment $l_{r}$; however, if $\theta_{r}>\theta_{\min}$ indicates that the resultant merged line segment diverges from the original, the merger is subsequently abandoned.

(5) Loop detection. The final step involves an iterative process where the algorithm repeatedly applies the above operations until no further mergers or eliminations are needed. This looping mechanism ensures the extraction process is thorough, yielding a refined set of line features that are both reliable and effective for use in SLAM. The continuous refinement enhances the robustness of the system, making it well suited for complex environments with varying textures and motion dynamics.

### 3.2. Line Feature Description and Matching

This article utilizes the LBD descriptor to conduct geometric consistency assessments of line features between consecutive frames based on the refined line feature extraction approach to improve matching precision. Spatial lines possess four degrees of freedom; however, the Plücker [23] parameterization approach employs six parameters to characterize a line, resulting in over-parameterization and heightened optimization complexity. This work presents an orthogonal representation that articulates lines using four parameters, streamlining the optimization process by lowering its dimensionality. The Plücker coordinates and orthogonal representation [24] can be easily interconverted, facilitating the utilization of both parameterization forms at various stages. Plücker coordinates are utilized in initialization and spatial transformation, while the orthogonal representation is applied in back-end optimization to enhance efficiency. The spatial line $l_{w}\in \pi_{w}$, located at points $c_{1}$ and $c_{2}$ in two camera frames and represented in Plücker coordinates as $l_{w}=\left(n_{w}^{T},d_{w}^{T}\right)$, has the following representation for the spatial line segment:

(5) $$\begin{array}{l} l_{w}^{*}=\left(\begin{array}{cc} \left[d_{w}\right] & n_{w}\\ -n_{w}^{T} & 0 \end{array}\right)\\ \pi_{1}=\left(c_{1},l_{w}\right),\pi_{2}=\left(c_{2},l_{w}\right) \end{array}$$

where $n_{w}$ represents the plane’s normal vector defined by $l_{w}$ and the origin of plane $\pi_{w}$; $d_{w}$ is the direction vector established by the two ends of $l_{w}$; $\pi_{1},\pi_{2}$ signifies the plane defined by the two places and $l_{w}$. To simplify the back-end optimization solution, the orthogonal representation method is employed in the optimization process. The Plücker coordinates are decomposed, and the orthogonal form of $l_{w}={\left(n_{w}^{T},d_{w}^{T}\right)}^{T}$ is denoted as $\left(U,W\right)\in SO\left(3\right)\times SO\left(2\right)$. The rotation between the line coordinate system and the camera coordinate system is specified as

(6) $$\begin{array}{l} \left[n_{w},d_{w}\right]=U\left[\begin{array}{cc} w_{1} & 0\\ 0 & w_{2}\\ 0 & 0 \end{array}\right]\\ W=\left(\begin{array}{cc} w_{1} & w_{2}\\ -w_{2} & w_{1} \end{array}\right) \end{array}$$

### 3.3. Reprojection Residual Model Construction

After reprojecting the line in space onto the image plane, compute the discrepancy between it and the corresponding line. Initially, execute a geometric transformation on the line feature and establish the transformation matrix $T_{cw}=\left[R_{cw},t_{cw}\right]$ from plane $\pi_{w}$ to plane $\pi_{c}$. $R_{cw}$ and $t_{cw}$ represent the designated line rotation and line translation, respectively. The matrix indicates that $l_{w}$ in $\pi_{w}$ can be transformed into $l_{c}$ using matrix transformation.

(7) $$l_{c}=\left[\begin{array}{c} n_{c}\\ d_{c} \end{array}\right]=\left[\begin{array}{cc} R_{cw} & \left[t_{cw}\right]\times R_{cw}\\ 0 & R_{cw} \end{array}\right]\left[\begin{array}{c} n_{w}\\ d_{w} \end{array}\right]$$

where $l_{c}$ represents the Plücker coordinate of $l_{w}$ within plane $\pi_{w}$. Subsequently, projecting $l_{c}$ onto the picture plane results in I, represented in Plücker coordinates as

(8) $$I={\left[l_{1},l_{2},l_{3}\right]}^{T}=K_{l}n_{c}$$

where $K_{l}$ denotes the linear projection matrix. Let $l_{w}$ denote the i camera frame $c_{i}$ and the j observed spatial line $\varsigma_{j}$; thus, the direct reprojection error is defined as

(9) $$r_{L}\left(z_{\varsigma_{j}}^{c_{i}},x\right)=d\left(m,I\right)=\frac{m^{T}I}{\sqrt{l_{1}^{2}+l_{2}^{2}}}\in {\mathbb{R}}^{1}$$

where $d\left(m,I\right)$ represents the distance function from a point to a line; m is the homogeneous coordinates at the mid-point of the line feature.

### 3.4. IMU Data Pre-Integration

Inertial Measurement Unit (IMU) sensors comprise a tri-axis gyroscope and a tri-axis accelerometer, enabling the acquisition of real-time angular velocity and acceleration data of the mobile robot during motion. Nonetheless, owing to intrinsic sensor bias and noise interference, IMU data frequently exhibit inaccuracies in actual applications [25]. Consequently, a measurement model incorporating noise and bias is generally employed to precisely characterize the IMU output. The mathematical representation of this measurement paradigm is as follows:

(10) $$\begin{array}{l} {\tilde{w}}_{b}^{W}\left(t\right)=w_{b}^{W}\left(t\right)+b_{g}\left(t\right)+\eta_{g}\left(t\right)\\ {\tilde{\alpha }}_{b}^{W}\left(t\right)={\left(R_{b}^{W}\right)}^{T}\left(\alpha_{b}^{W}\left(t\right)-g^{w}\right)+b_{\alpha }\left(t\right)+\eta_{\alpha }\left(t\right) \end{array}$$

The expression for the motion model of the IMU is derived as follows:

(11) $${\bar{q}}_{b}^{W}=q_{b}^{W}⊗\left(\begin{array}{c} 0\\ \frac{1}{2}w_{b}^{W} \end{array}\right),{\bar{v}}_{b}^{W}=\alpha_{b}^{W},{\bar{p}}_{b}^{W}=v_{b}^{W}$$

**Figure 4:**
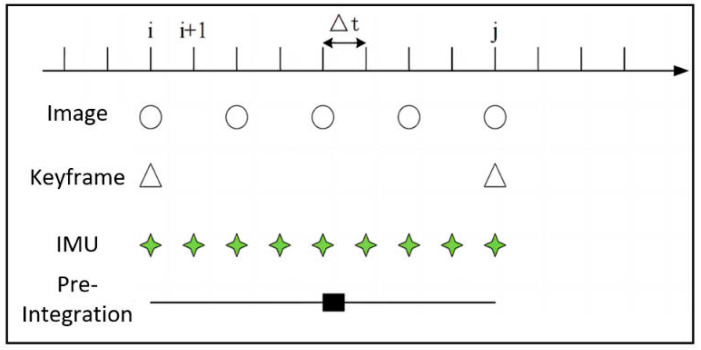
Visual observation model and IMU schematic diagram. The IMU data must be integrated and calculated in discrete time due to the fact that its data acquisition frequency is significantly higher than that of the camera. Consequently, a unified data format is necessary to ensure close coupling of the data. This diagram uses hollow circles, hollow triangles, green stars, and black squares to represent image frames, keyframes, IMU data, and the pre-integration process.

In actual implementations, the IMU’s data acquisition frequency significantly exceeds the camera’s, as illustrated in Figure 4, necessitating a uniform data format for data fusion. The method employs a tightly coupled framework to synchronize IMU data with visual data. Given the high sampling frequency of the IMU, it provides dense motion information compared to camera data. An IMU pre-integration approach is used to process data between keyframes. Specifically, the IMU data accumulated between two consecutive keyframes are pre-integrated to calculate changes in orientation, velocity, and position, providing accurate motion estimation. Simultaneously, visual data are used to detect and match feature points at keyframes, forming visual constraints. By integrating IMU pre-integration residuals and visual observation residuals, a nonlinear optimization problem is formulated using a sliding window technique. This approach adjusts IMU bias parameters, scale factors, and camera poses, while also refining the positions of visual map points. The tightly coupled strategy significantly enhances pose estimation accuracy and system synchronization in complex motion scenarios. This research employs a mid-point integration method to enhance the accuracy of IMU data processing. For the position variation between frame k and frame k+1, the median of the IMU data gathered between these two frames is utilized for estimation. The average of all IMU measurements between the time i and i+1 is computed to estimate the pose change between successive time intervals, thereby mitigating the error accumulation caused by IMU noise.

(12) $$\begin{array}{l} p_{b_{i+1}}^{W}=p_{b_{i}}^{W}+v_{b_{i}}^{W}\delta t+\frac{1}{2}{\bar{\tilde{\alpha }}}_{\iota }t^{2}\\ v_{b_{i+1}}^{W}=v_{b_{i}}^{W}+{\bar{\tilde{\alpha }}}_{\iota }\delta t\\ q_{b_{i+1}}^{W}=q_{b_{i}}^{W}⊗\left(\begin{array}{c} 0\\ \frac{1}{2}{\bar{\tilde{w}}}_{\iota }\delta t \end{array}\right) \end{array}$$

The necessity of integrating all IMU data to calculate the pose for each new image frame escalates computational complexity and impacts the system’s real-time performance. To enhance computational efficiency, the integration value is revised by computing the incremental variations in rotation, velocity, and displacement between two frames, circumventing superfluous calculations for each measurement and significantly lowering computational expenses. IMU pre-integration not only reduces computational demands but also maintains the precision of sensor data. In the continuous-time model, the pre-integrated value is exclusively contingent upon the IMU measurements at various time intervals and their associated biases. According to the mid-point integration approach, the IMU pre-integration value in discrete time is articulated as follows:

(13) $$\begin{array}{l} {\tilde{\alpha }}_{i+1}^{b_{k}}={\tilde{\alpha }}_{i}^{b_{k}}+\beta_{i}^{b_{k}}\delta t+\frac{1}{2}{\bar{\tilde{\alpha }}}_{\iota }\delta t^{2}\\ {\tilde{\beta }}_{i+1}^{b_{k}}={\tilde{\beta }}_{i}^{b_{k}}+{\bar{\tilde{\alpha }}}_{\iota }\delta t\\ {\tilde{\gamma }}_{i+1}^{b_{k}}={\tilde{\gamma }}_{i}^{b_{k}}⊗{\tilde{\gamma }}_{i+1}^{i}={\tilde{\gamma }}_{i}^{b_{k}}⊗\left(\begin{array}{c} 0\\ \frac{1}{2}{\bar{\tilde{w}}}_{\iota }\delta t \end{array}\right) \end{array}$$

where ${\tilde{\alpha }}_{i+1}^{b_{k}},{\tilde{\beta }}_{i+1}^{b_{k}}$, and ${\tilde{\gamma }}_{i+1}^{b_{k}}$ denote the pre-integrated values of the IMU that require resolution. In the back-end optimization phase of pose estimation utilizing IMU data, the implementation of the IMU pre-integration method can substantially diminish redundant computations between frames, thereby significantly reducing the overall computational complexity of the SLAM system and improving its operational efficiency.

## 4. Back-End Optimization and Loop Closure Detection

### 4.1. Multi-Source Fusion Sliding Window Optimization

A multi-source information fusion sliding window nonlinear optimization framework is implemented in the back-end optimization of the visual–inertial SLAM system to guarantee real-time performance and robustness. The sliding window approach dynamically modifies the inclusion or exclusion of optimization variables, enabling keyframes within a specified time window to significantly enhance the pose estimation process. This dramatically decreases computing complexity while maintaining accuracy. The sliding window model incorporates the complete state vector χ, encompassing point features, line features, and IMU data, articulated as follows:

(14) $$\begin{array}{l} \chi =\left[x_{0},x_{1},\cdots ,x_{n_{k}},\lambda_{0},\lambda_{1},\cdots ,\lambda_{n_{p}},{ο}_{0},{ο}_{1},\cdots ,{ο}_{n_{l}}\right]\\ x_{k}=\left[p_{b_{k}^{w}},q_{b_{k}^{w}},v_{b_{k}^{w}},b_{a},b_{g}\right],k\in \left[0,n_{k}\right] \end{array}$$

The data in $x_{k}$ include the position $p_{b_{k}^{w}}$, direction $q_{b_{k}^{w}}$, and velocity $v_{b_{k}^{w}}$ of the IMU in the k frame, along with the IMU accelerometer bias $b_{a}$ and gyroscope $b_{g}$. The subscripts $n_{k},n_{p}$, and $n_{l}$ denote the quantity of keyframes, the count of point features within the keyframes, and the number of line features in the keyframes, respectively. $\lambda_{n_{p}}$ represents the inverse depth of the three-dimensional point, while ${ο}_{n_{p}}$ signifies the four-parameter orthogonal representation of the three-dimensional line.

(15) $$\underset{\chi }{\min}\left\{\begin{array}{l} \rho_{c}\left({\left\|r_{p}-J_{p}\chi \right\|}^{2}\right)+\sum_{k\in B}\rho_{c}\left({\left\|r_{B}\left(z_{b_{k+1}}^{b_{k}},\chi \right)\right\|}_{p_{b_{k+1}}^{b_{k}}}^{2}\right)+\\ \sum_{\left(f,i\right)\in F}\rho_{h}\left({\left\|r_{F}\left(z_{f}^{c_{j}},\chi \right)\right\|}_{p_{f}^{c_{j}}}^{2}\right)+\sum_{\left(l,j\right)\in L}\rho_{h}\left({\left\|r_{L}\left(z_{l}^{c_{j}},\chi \right)\right\|}_{p_{l}^{c_{j}}}^{2}\right) \end{array}\right\}$$

where $r_{B}\left(z_{b_{k+1}}^{b_{k}},\chi \right)$ denotes the IMU residual between frames $x_{k}$ and $x_{k-1}$, B represents the collection of IMU pre-integration measurement values, $r_{F}\left(z_{f}^{c_{i}},\chi \right)$ signifies the reprojection residual of point features, $r_{L}\left(z_{l}^{c_{j}},\chi \right)$ indicates the reprojection residual of line features, and F and L are the sets of point and line features in the image, respectively. $p_{b_{k+1}}^{b_{k}},p_{f}^{c_{i}}$, and $p_{l}^{c_{j}}$ are the covariance matrices corresponding to IMU pre-integration residuals, point feature residuals, and line feature residuals, respectively. $r_{p}$ refers to the prior residual, $J_{p}$ is the Jacobian matrix of the prior residual, $\rho_{c}$ is the Cauchy robust kernel function for outlier elimination, and $\rho_{h}$ is the Huber kernel function for minimizing the mismatch rate.

### 4.2. Marginalization Model

Mainstream VIO systems generally utilize sliding window and marginalization techniques to decrease the number of optimization parameters, thus reducing computational complexity and enhancing the accuracy and efficiency of state estimation. In the back-end optimization process, marginalization separates the joint probability distribution into marginal and conditional distributions, minimizing unnecessary computational burden. The marginalization model presented in this research functions within a fixed-size sliding window, improving real-time speed and robustness. By excluding the oldest or second-oldest frame within the sliding window while maintaining its constraints with other keyframes, the method eliminates duplicate calculations for pose and associated feature points, minimizing the state variables. This approach effectively tackles optimization issues in compromised situations, preserving global restrictions while enhancing the efficiency of back-end optimization and the system’s overall correctness and stability.

The Gauss–Newton iteration can be employed to solve the nonlinear least squares problem in the optimization process, as illustrated by the following: Hδx=b. The Hδx=b formula can be expressed as follows when the marginalized state variable is set to $\chi_{a}$ and the retained variable is set to $\chi_{b}$ in this paper:

(16) $$\left[\begin{array}{cc} \Lambda_{a} & \Lambda_{b}\\ \Lambda_{b}^{T} & \Lambda_{c} \end{array}\right]\left[\begin{array}{c} \delta \chi_{a}\\ \delta \chi_{b} \end{array}\right]=\left[\begin{array}{c} g_{a}\\ g_{b} \end{array}\right]$$

The prior information of variable $\delta \chi_{b}$ can be determined by employing the Schur complement method to deduce the following formula:

(17) $$\left[\begin{array}{cc} \Lambda_{a} & \Lambda_{b}\\ 0 & \Lambda_{c}-\Lambda_{b}^{T}\Lambda_{a}^{-1}\Lambda_{b} \end{array}\right]\left[\begin{array}{c} \delta \chi_{a}\\ \delta \chi_{b} \end{array}\right]=\left[\begin{array}{c} g_{a}\\ g_{b}-\Lambda_{b}^{T}\Lambda_{a}^{-1}g_{a} \end{array}\right]$$

(18) $$\left(\Lambda_{c}-\Lambda_{b}^{T}\Lambda_{a}^{-1}\Lambda_{b}\right)\delta \chi_{b}=g_{b}-\Lambda_{b}^{T}\Lambda_{a}^{-1}g_{a}$$

**Figure 5:**
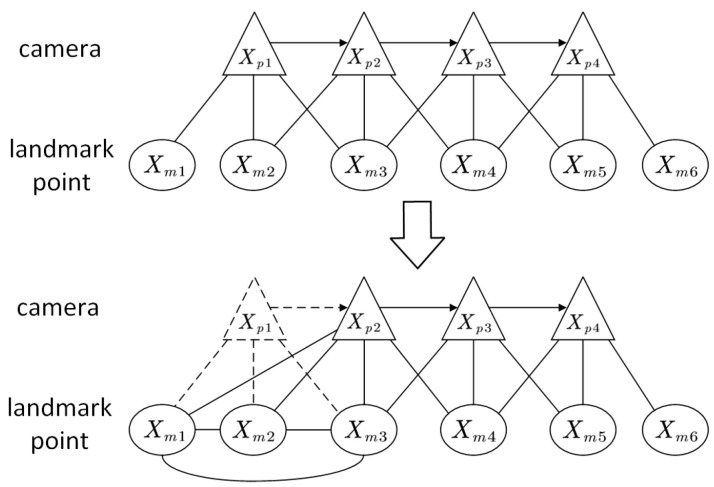
Marginalization model. The relationship model between the camera and the landmark locations during the marginalization process.

Figure 5 illustrates the relationship model between the camera and the landmark locations during the marginalization process. The marginalized variable equation is $\delta \chi_{a}=\delta \chi_{p_{1}}^{6\times 1}$ when $X_{p_{1}}$ is marginalized. Consequently, new constraints will be established between $X_{m_{1}}$, $X_{m_{2}}$, and $X_{m_{3}}$, as well as between $X_{m_{1}}$ and $X_{p_{2}}$. At present, $\Lambda_{c}-\Lambda_{b}^{T}\Lambda_{a}^{-1}\Lambda_{b}$ corresponds to the information matrix H. Then, $X_{m_{1}}$ continues to be marginalized in a manner that is similar.

### 4.3. Point–Line Fusion Loop Closure Detection

Loop closure detection is essential for achieving globally consistent posture estimation in the SLAM system. Loop closure delivers local restrictions between neighboring frames and creates long-term associations with prior frames, providing broader constraints that enhance the system’s accuracy and robustness throughout prolonged operations.

This research presents an approach that employs a loop closure detection framework utilizing the bag-of-words model [25], which analyzes both keypoint and key line properties for loop closure detection. Upon identifying a keyframe, the system encodes point and line features utilizing ORB descriptors and LBD descriptors, respectively, producing corresponding word vectors. For keyframe $s_{k}$, which encompasses both point and line characteristics, $s_{k}=\omega_{p}s_{p}+\omega_{l}s_{l}$ is delineated as follows:

(19) $$\begin{array}{l} \omega_{p}=\frac{1}{2}\left(\frac{n_{p}}{n_{p}+n_{l}}+\frac{d_{p}}{d_{p}+d_{l}}\right)\\ \omega_{l}=\frac{1}{2}\left(\frac{n_{l}}{n_{p}+n_{l}}+\frac{d_{l}}{d_{p}+d_{l}}\right) \end{array}$$

The loop closure detection technique initially gathers corner information from the current frame and produces descriptors for matching purposes. The system subsequently searches for loop closure candidate frames inside the keyframe database. Upon detecting a loop closure, the system delivers relevant loop closure constraints and integrates them into the objective function for nonlinear optimization to resolve the relative pose connections among frames. Consequently, all frames within the sliding window are globally calibrated for accurate pose correction. Loop closure optimization rectifies the pose of the current frame while also optimizing and integrating historical pose data into the global map, ensuring consistency of the global map and preserving high localization accuracy across extended operations.

## 5. Experimental Design and Results Analysis

This study performs trials on the EuRoC dataset and simulated open-pit mining conditions to assess the efficacy of the proposed method in intricate situations. The experiment has two segments: Initially, the enhanced line feature extraction and matching algorithm presented in this research is assessed against conventional LSD and EDLines methods to determine their efficacy in feature extraction. The effectiveness of the suggested approach is evaluated in comparison to VINS-Mono, ORB-SLAM3, and PL-VIO algorithms using both public datasets and simulated open-pit mining contexts. VINS-Mono and ORB-SLAM3, as leading visual–inertial SLAM systems, utilize point characteristics for front-end tracking, whereas the PL-VIO method enhances adaptation to intricate situations by using point and line information. Nonetheless, PL-VIO experiences significant computational complexity in line feature extraction and matching, which may introduce computational overhead to real-time systems. Experimental results indicate that the enhanced method markedly improves accuracy in global trajectory estimation and further illustrates the superiority of the proposed point–line fusion visual–inertial SLAM technique in terms of accuracy and robustness.

### 5.1. Comparative Analysis of Enhanced Line Feature Extraction Algorithm

To assess the efficacy of the enhanced line feature extraction algorithm, we choose the MH_04_difficult, MH_05_difficult, V1_03_difficult, and V2_03_difficult sequences from the EuRoC dataset, together with two simulated open-pit mining situations (elaborated in Section 4.3) for evaluation. This sequence comprises scenes characterized by rapid motion, inadequate lighting, and diverse texture quality, facilitating a comprehensive assessment of the algorithm’s robustness and efficacy in intricate settings. In the studies, we evaluate the effectiveness of the EDLines algorithm, recognized for its rapid line identification in SLAM systems, against the original and enhanced LSD algorithms introduced in this study. The mean processing duration per frame and the quantity of extracted line features are documented, as illustrated in Table 1. The enhanced LSD algorithm incorporates segment filtering and nearby line merging techniques derived from parameter optimization.

**Figure 6:**
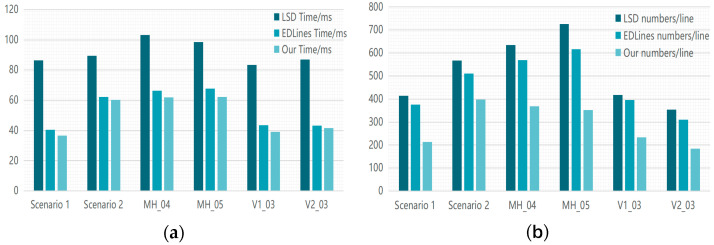
Histogram for the performance comparison of the line feature extraction algorithm: (**a**) the average time required to derive line features; (**b**) the average number of line feature extractions.

Experimental results indicate that the improved LSD method decreases extraction time by roughly 45.34% on average (refer to Figure 6a). Still, the quantity of retrieved line features diminishes by an average of 44.32% (refer to Figure 6b). Compared to the EDLines method, the enhanced LSD algorithm decreases the number of line features by roughly 37.46% while maintaining the long line features essential for localization accuracy and significantly reducing processing time. The suggested enhanced approach markedly increases the quality of line feature extraction, mitigates the adverse effects of irrelevant features on SLAM system accuracy, and elevates overall system performance.

**Figure 7:**
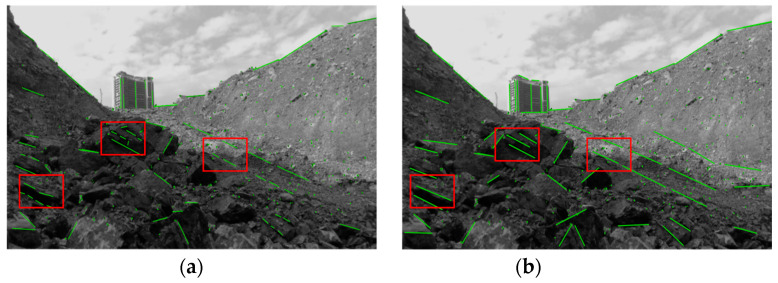
Line feature extraction algorithm performance comparison: (**a**) the LSD algorithm’s effect on line feature extraction; (**b**) the enhanced LSD method. Utilizing short segment elimination and approximate line merging techniques markedly eliminates redundant short line features while preserving the longer line segments essential for localization precision. The red box highlights the comparison section between the two images, with the green dots and lines representing the extracted point and line features from the images, respectively.

Figure 7 illustrates the feature extraction performance of the LSD algorithms before and after enhancements. Subfigures (a) and (b) present the test outcomes from Dataset Sequence 2 in a practical open-pit mining context. The original LSD algorithm generates numerous short and comparable line segments, augmenting the computational load of line identification and matching while introducing ambiguity in the system’s localization precision. The enhanced LSD method, utilizing short segment elimination and approximate line merging techniques, markedly eliminates redundant short line features while preserving the longer line segments essential for localization precision. This enhancement improves the quality of line feature extraction, hence significantly augmenting the localization accuracy and general robustness of the SLAM system.

The experiments’ results confirm that the enhanced LSD algorithm markedly decreases line extraction duration while improving the quality of long line characteristics, hence ensuring both real-time efficacy and robustness. The line feature extraction technique presented in this research exhibits enhanced performance relative to conventional algorithms in intricate illumination and dynamic motion settings. This enhancement substantially optimizes the system’s back-end processing and multi-sensor data fusion, significantly contributing to the overall localization accuracy and stability of the system.

### 5.2. Evaluation of System Accuracy

In mobile robot SLAM systems, Absolute Pose Error (APE) and Relative Pose Error (RPE) are essential metrics for assessing localization accuracy. Among these, the absolute trajectory error (ATE) immediately assesses the discrepancy between actual and estimated trajectories in three-dimensional space, rendering it one of the most prevalent and intuitive approaches for evaluating localization mistakes. To determine the robustness and efficacy of the proposed algorithm, numerous comparative experiments are performed utilizing the publicly accessible EuRoC dataset, employing the Root Mean Square Error (RMSE) for quantitative evaluation of the algorithm’s performance relative to prominent visual SLAM algorithms. Table 2 delineates the quantitative evaluation findings utilizing the APE metric, juxtaposing the efficacy of the suggested technique against systems such as VINS-Mono, PL-VIO, and ORB-SLAM3, thus offering a definitive appraisal of the proposed algorithm’s superiority.

Table 2 illustrates that the suggested technique, which integrates line characteristics, markedly enhances localization accuracy compared to visual SLAM algorithms that depend exclusively on point features. Through the enhancement of the LSD algorithm, high-quality line features are efficiently retrieved, and the suggested method surpasses the newest PL-VIO methodology across most dataset sequences, thereby boosting localization accuracy and decreasing computing time.

In contrast to VINS-Mono, which excels in high-texture situations, its accuracy markedly declines in low-texture scenarios due to a lack of adequate feature points, as demonstrated by an RMSE of 0.351601 m in MH_04_difficult. While PL-VIO demonstrates stability across many illumination situations, its elevated computational complexity constrains its efficacy in dynamic or low-feature settings. For example, despite attaining an RMSE of 0.084627 m in the V1_01_easy sequence, there remains potential for enhancement in more intricate scenarios. ORB-SLAM3 often exhibits commendable performance; nevertheless, it encounters difficulties with significant lighting fluctuations and intricate terrain, resulting in an RMSE of 0.067150 m in MH_05_difficult. Conversely, our approach decreases the RMSE to 0.046667 m in identical circumstances, signifying a 30.5% enhancement in precision. Our method substantially improves resilience and stability in situations with extreme illumination variations and low texture by integrating enhanced LSD algorithms with line characteristics. In the V2_03_difficult sequence, the RMSE diminishes to 0.125673 m, reflecting a 28.7% decrease relative to ORB-SLAM3. Compared to ORB-SLAM3, the proposed approach enhances RMSE accuracy by 36.62% and 26.88% in the MH and VR sequences, respectively. In intricate environments, such as the MH_04_difficult, MH_05_difficult, and V2_03_difficult sequences, marked by substantial lighting variations and limited features, the proposed algorithm demonstrates remarkable robustness without any feature loss, showcasing the efficacy of the enhanced line feature extraction and matching algorithm in demanding situations.

**Figure 8:**
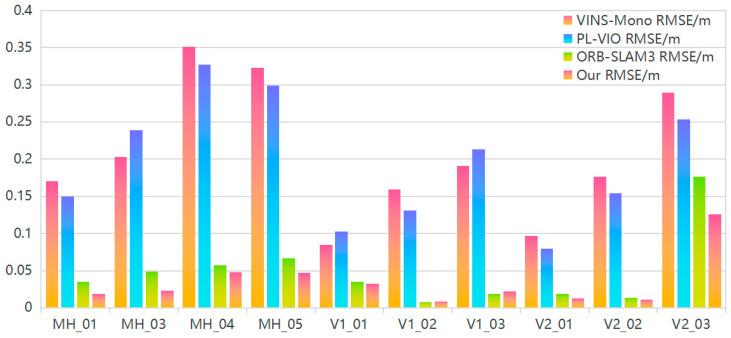
Histogram of absolute trajectory error. The histogram illustrates that, in the MH sequence, the absolute trajectory error of the enhanced algorithm is less than that of other algorithms, whereas, in the VR sequence, the enhanced algorithm performs comparably to or better than the perfect algorithm.

The RMSE results of several techniques are illustrated in histogram format to elucidate the absolute trajectory errors (refer to Figure 8). The figure indicates that the suggested approach exhibits superior accuracy and robustness in these intricate settings. In the MH sequences, data are acquired by a drone in low-texture situations characterized by intricate lighting conditions, akin to the difficulties of feature point extraction from rocky terrains in open-pit mining settings. The VR scenes are captured with a handheld camera inside, where point and line features are intricate and characterized by significant irregular, high-velocity motion, analogous to the feature extraction loss experienced on uneven terrain in open-pit mines. The findings validate that the suggested system exhibits exceptional robustness and superior localization accuracy, particularly in intricate situations like open-pit mines.

To assess the stability of the proposed approach relative to conventional localization algorithms, trajectory error comparisons are performed utilizing the EuRoC dataset on the MH_04_difficult and V2_03_difficult sequences in comparison with the enhanced ORB-SLAM3, VINS-Mono, and PL-VIO traditional algorithms.

**Figure 9:**
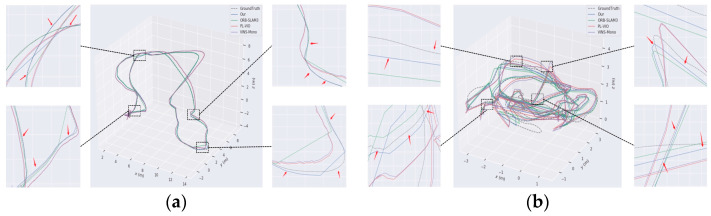
Analysis results of trajectory error comparison: (**a**) comparison of the trajectory for Sequence MH_04_difficult; (**b**) comparison of difficult trajectories in Sequence V2_03. The black boxes and red arrows in the figure are used to enlarge key areas and mark trajectory deviations, highlighting the accuracy differences among different algorithms in these regions.

The comparison results are shown in Figure 9, where the dashed line represents the actual trajectory (ground truth), the blue curve denotes the estimated trajectory of the proposed algorithm, the green curve corresponds to the original ORB-SLAM3 algorithm, the red curve represents the PL-VIO algorithm, and the purple curve indicates the VINS-Mono algorithm. From the red arrows in the magnified sections on the left and right sides of the figure, it is evident that even under conditions of rapid direction changes, motion blur, and poor texture quality, the proposed algorithm closely aligns with the actual trajectory. Specifically, in the MH_04_difficult scenario, ORB-SLAM3 and VINS-Mono exhibit significant trajectory deviations, while in the V2_03_difficult scenario, the four sharp turns highlighted in the magnified images reveal that PL-VIO’s trajectory shows the largest deviations and highest errors compared to the actual path. In contrast, the proposed algorithm demonstrates the best alignment with the ground truth, showcasing superior stability and robustness in low-texture and high-motion environments. By incorporating line features, the proposed algorithm significantly enhances pose estimation accuracy, achieving a high degree of consistency with the actual trajectory and meeting the stringent requirements of SLAM systems for precision and robustness.

**Figure 10:**
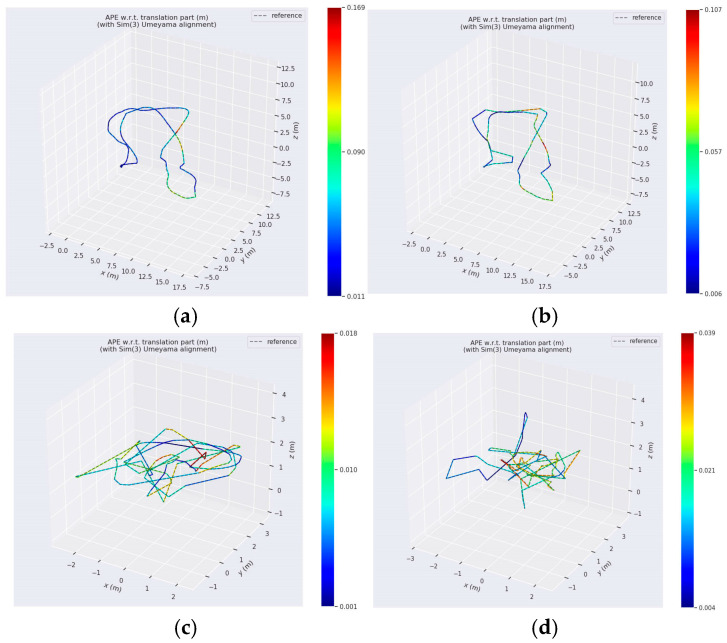
Results of absolute pose inaccuracy for each series: (**a**) Sequence MH_04_difficult; (**b**) Sequence MH_05_difficult; (**c**) Sequence V1_02_medium; (**d**) Sequence V1_03_difficult. The color-coded line represents varying levels of Absolute Pose Error (APE) along the trajectory, with red indicating higher error and blue indicating lower error, highlighting accuracy differences across segments.

Figure 10 illustrates an intuitive comparison between the estimated trajectory of the proposed algorithm (solid line) and the ground truth trajectory (dashed line) for a variety of sequences, such as MH_04_difficult, MH_05_difficult, V1_02_medium, and V2_03_difficult. The error range is denoted by a gradient color bar that transitions from deep blue to red, representing the distribution of errors from minimal to maximal. The results indicate that the proposed algorithm consistently accomplishes exceptional stability and low error levels across all sequences, particularly in MH_05_difficult, where it maintains minimal pose estimation errors even under complex lighting variations. The method’s exceptional stability and accuracy in challenging environments are underscored by this performance, which effectively manages errors within acceptable limits in scenarios that involve acceleration, abrupt turns, and drastic lighting changes. Additionally, the proposed algorithm demonstrates considerable robustness in the face of external disturbances and abrupt direction changes in V1_02_medium and V2_03_difficult sequences, despite the increased motion dynamics. In summary, the system’s superior performance in terms of both accuracy and robustness is confirmed by its exceptional pose estimation capabilities in complex environments.

**Figure 11:**
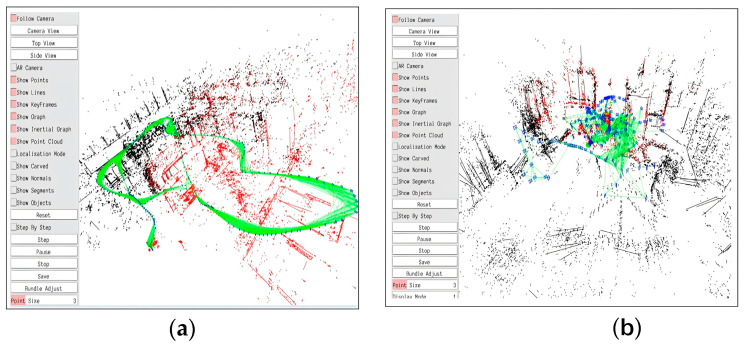
Three-dimensional point cloud maps: (**a**) Sequence MH_05_difficult; (**b**) Sequence V1_03_difficult. The figure shows a 3D mapping visualization where the green lines represent the estimated trajectory, red points indicate mapped features, and black points show additional environmental points.

Figure 11 illustrates the real-time trajectory and the three-dimensional line feature map produced by the system throughout its operation. The algorithm’s pose estimation accuracy is evaluated using the MH_05_difficult dataset, noted for its low-texture environment, and the V1_03_difficult dataset, which features considerable viewpoint jitter. Experimental results indicate that line feature extraction is highly efficient, and the mapping of line features displays significant robustness. The system effectively creates a high-precision point–line structure map applicable to many difficult circumstances, demonstrating the algorithm’s adaptability and accuracy across multiple environments.

### 5.3. Real-World Localization Experiment Validation

To ascertain the feasibility of the proposed localization method in practical applications and to simulate an open-pit mining environment, data are gathered from a simulated open-pit mine characterized by sparse feature points on rugged terrain and an uneven road with considerable gradient. The experiment is structured to evaluate the system’s localization accuracy on linear lines and curved parts, categorized into Scenario 1 and Scenario 2.

**Figure 12:**
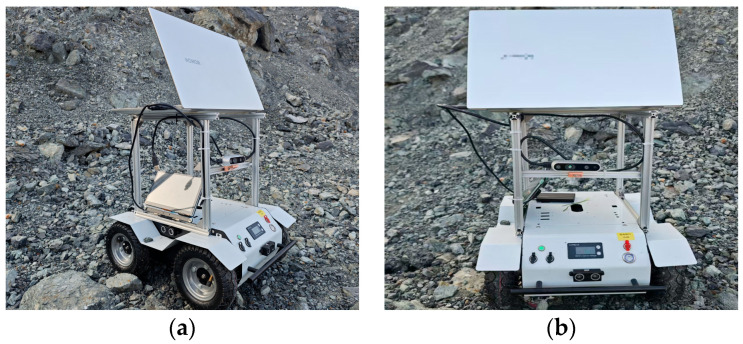
Experimental mining intelligent robot platform: (**a**) left view; (**b**) front view.

The experimental platform utilizes a 64-bit Linux operating system, operating on an Intel Core™ i7-10510U processor (four cores) with 8GB of RAM. The used camera is the Intel RealSense Depth Camera D435i. The operating system utilized is Ubuntu 20.04, and the algorithm is executed in the Noetic version of the Robot Operating System (ROS). The mining intelligent robot platform, depicted in Figure 12, is effectively utilized for data collecting and system validation in experimental settings.

**Figure 13:**
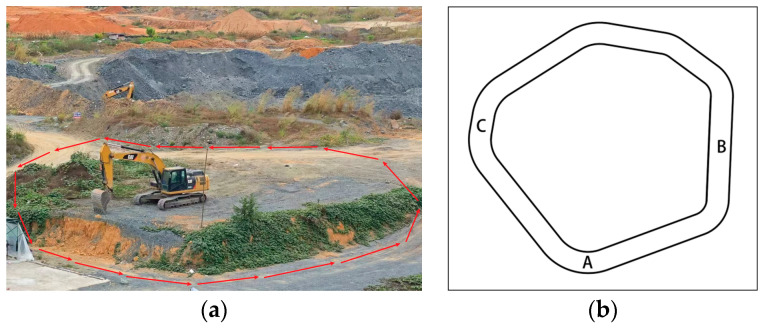
Scene 1: circular open-pit excavation: (**a**) real-world scene; (**b**) diagram of movement trajectory. In (**a**), the red arrows represent the motion trajectory of the mapping robot. In (**b**), points A, B, and C represent key checkpoints along the closed-loop path.

**Figure 14:**
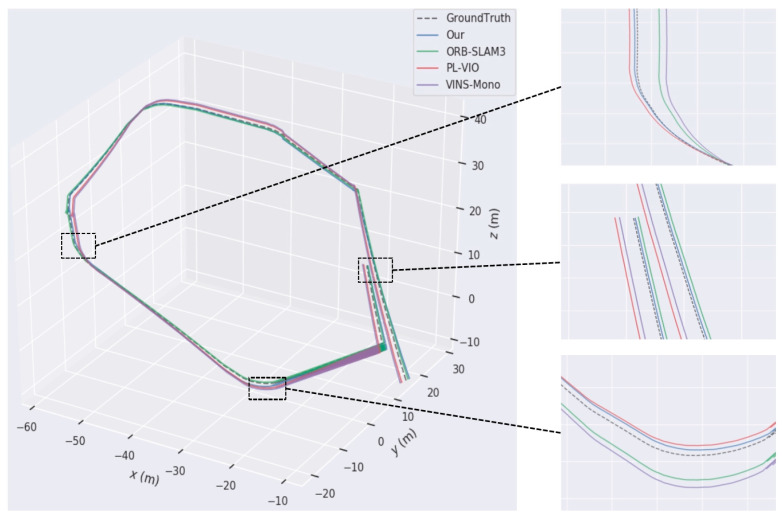
Comparison of trajectory errors in Scenario 1.

In the circular simulated open-pit mining site of Scenario 1, seen in Figure 13, points A and C on the designated rough surfaces exhibit sparse feature points, which may result in tracking loss. Both the PL-VIO algorithm and the proposed approach incorporate line feature aid within the feature extraction module to improve feature extraction efficacy. In comparison to PL-VIO, the enhanced line feature extraction algorithm presented in this paper markedly enhances feature point completion in sparse environments, resulting in an estimated trajectory that more closely aligns with the actual trajectory, thus augmenting trajectory estimation accuracy (refer to the trajectory error comparison at points A and C in Figure 14). Point B is a steep incline, where swift visual alterations from elevation and irregularities may result in transient feature point loss. The proposed technique and ORB-SLAM3 ensure consistent localization in these settings via robust visual–inertial integration. Experimental results indicate that the suggested algorithm displays considerable robustness during the experiment, with system stability and robustness markedly exceeding those of the comparative algorithms, resulting in concurrent enhancements in accuracy and robustness.

**Figure 15:**
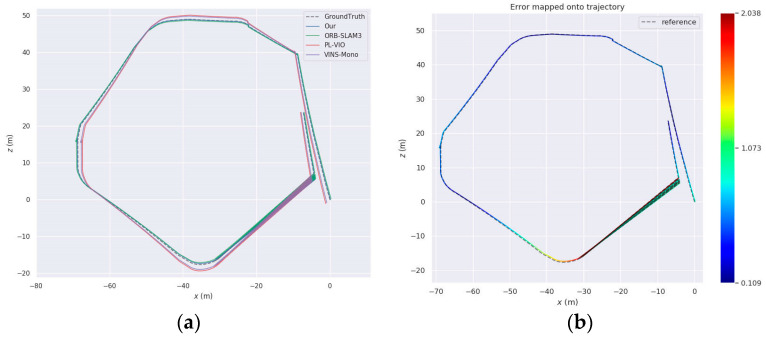
Analysis of experimental outcomes in Scenario 1: (**a**) comparison of 2D plane trajectories; (**b**) absolute trajectory error of data series.

Figure 15 presents subfigures (a) and (b), which illustrate the comparison of planar trajectory error and the graph of absolute trajectory error, respectively. The projected trajectory of the proposed enhanced algorithm aligns much better with the actual trajectory, indicating its increased localization accuracy.

Table 3 demonstrates that the Root Mean Square Error (RMSE) and mean value analysis based on absolute trajectory error (ATE) reveal that the proposed algorithm consistently yields the lowest error values in the test scenarios, attaining the highest localization accuracy and closely approximating the ground truth. Compared to ORB-SLAM3, the enhanced point–line feature matching technique presented in this research increases localization accuracy by an average of 40.62% in Scenario 1, substantiating the algorithm’s efficacy and dependability in intricate situations.

**Figure 16:**
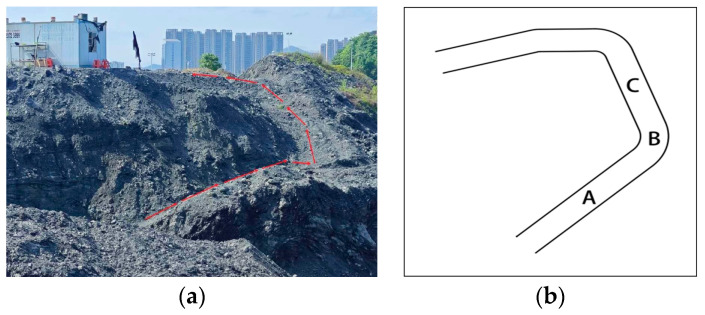
Scene 2: Uneven road conditions in an open-pit mine: (**a**) real-world scene; (**b**) diagram of movement trajectory. In (**a**), the red arrows represent the motion trajectory of the mapping robot. In (**b**), points A, B, and C represent key checkpoints along the path.

**Figure 17:**
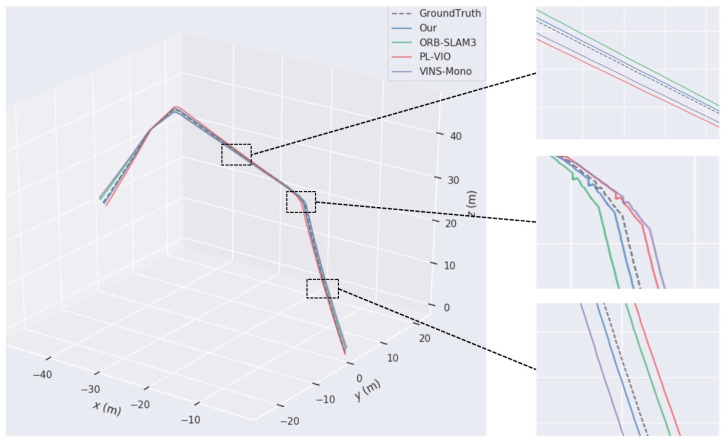
Comparison of trajectory errors in Scenario 2.

Scenario 2 features a testing environment characterized by a steep and uneven road, as illustrated in Figure 16. While navigating, the intelligent robot encounters considerable pitch and oscillation. From points A and C depicted in Figure 17, it is evident that the localization trajectories of the ORB-SLAM3 and VINS-Mono algorithms, both utilizing visual–inertial support, closely approximate the actual trajectory. In contrast, the estimated trajectory of the proposed algorithm exceeds both, demonstrating that localization algorithms with inertial support on uneven terrain can significantly mitigate drift and enhance localization precision. Furthermore, significant trajectory changes are observed at point B, where a severe turn exists. In this context, the trajectory estimations of the PL-VIO and the proposed approach, both utilizing point–line features, are more aligned with the actual trajectory, demonstrating that the enhanced line feature extraction and matching technique is superior in complicated circumstances.

The experiment illustrates that the enhanced method presented in this research attains a more consistent localization trajectory aligned with the actual operational trajectory in Scenario 2, thereby satisfying the high-precision localization demands for robots in open-pit mines. To quantitatively assess the localization accuracy of the system, absolute trajectory error (ATE) is evaluated using the Root Mean Square Error (RMSE) and mean value, as presented in Table 4. The enhanced point–line feature matching method increases localization accuracy by an average of 61.32% relative to the original ORB-SLAM3 system in Scenario 2, thereby confirming the algorithm’s efficacy and superiority in intricate situations.

## 6. Conclusions

(1) This study presents an improved visual–inertial real-time SLAM algorithm based on the ORB-SLAM3 framework, aimed at addressing critical challenges in open-pit mining environments, such as poor line feature extraction quality and significant jitter. By integrating point and line features in the front-end module and employing an enhanced LSD algorithm, the method significantly improves feature extraction accuracy and robustness. Additionally, a tightly coupled visual–inertial optimization model with a sliding window is implemented in the back-end to ensure reliable and precise pose estimation. Experimental results demonstrate that the enhanced algorithm achieves RMSE accuracy improvements of 36.62% and 26.88% on the MH and VR sequences of the EuRoC dataset, respectively. In real open-pit mining scenario tests, the modified algorithm greatly enhances feature extraction richness and reduces trajectory drift, with localization accuracy improvements of 40.62% and 61.32% in Scenario 1 and Scenario 2, respectively, compared to the original ORB-SLAM3 system. These advancements provide an efficient and reliable solution for real-time state estimation and environmental perception in complex open-pit mining environments, showcasing substantial potential for practical applications and future development.

(2) Nevertheless, this study has some limitations. The SLAM algorithm still falls short in handling dynamic environmental changes and unpredictable external interferences during actual operation. While this research primarily focuses on terrain irregularities, additional complexities need to be addressed in future studies. Furthermore, the lack of comprehensive comparisons with the latest methods optimized for similar environments limits a deeper understanding of the overall performance of the proposed approach. Future research will aim to integrate more advanced sensor fusion technologies, adopt machine learning models to predict and adapt to dynamic changes, or improve real-time filtering techniques. Additionally, systematic comparisons with the latest optimized methods will be conducted to enhance the system’s robustness, better support robotic path planning and navigation, and improve accuracy and stability in practical applications.

## 7. Patents

This research includes a patent titled “A Robot Localization and Mapping System”, with the publication number CN118342472A.

## Figures and Tables

**Table 1:** Results of performance comparison for line feature extraction techniques.

Sequence	LSD		EDLines		Ours	
	Numbers/Line	Time/ms	Numbers/Line	Time/ms	Numbers/Line	Time/ms
Scenario 1	415	86.5	375	40.6	213	36.7
Scenario 2	568	89.4	510	62.3	398	60.4
MH_04	635	103.3	569	66.2	368	61.9
MH_05	726	98.5	617	67.8	353	62.3
V1_03	418	83.4	395	43.5	233	39.1
V2_03	354	87.1	311	43.1	185	41.7

**Table 2:** Comparison of the absolute trajectory errors of four algorithms.

Sequence	VINS-Mono		PL-VIO		ORB-SLAM3		Ours	
	RMSE/m	Mean/m	RMSE/m	Mean/m	RMSE/m	Mean/m	RMSE/m	Mean/m
MH_01	0.170410	0.144947	0.149992	0.124888	0.035390	0.029830	**0.019034**	**0.016230**
MH_03	0.203385	0.187061	0.238910	0.219831	0.048817	0.044774	**0.023251**	**0.020838**
MH_04	0.351601	0.322701	0.326813	0.299383	0.057246	0.04934	**0.048088**	**0.042501**
MH_05	0.323365	0.304474	0.298774	0.281060	0.067150	0.060276	**0.046667**	**0.041904**
V1_01y	0.084627	0.075022	0.103103	0.092958	0.034904	0.031758	**0.032795**	**0.029826**
V1_02	0.159020	0.150049	0.131306	0.118482	**0.007813**	**0.007217**	0.008568	0.007788
V1_03	0.190584	0.173283	0.213054	0.193671	**0.018956**	**0.017240**	0.022056	0.020778
V2_01	0.097043	0.087504	0.079954	0.074809	0.018472	0.014673	**0.012600**	**0.011401**
V2_02	0.176523	0.150649	0.153797	0.140683	0.013429	0.012363	**0.010747**	**0.009912**
V2_03	0.289265	0.276645	0.253488	0.240798	0.176151	0.144637	**0.125673**	**0.091029**

The bold text in the table represents the minimum absolute trajectory error in each dataset sequence.

**Table 3:** The absolute trajectory error of various algorithms in Scenario 1.

Category	VINS-Mono	PL-VIO	ORB-SLAM3	Ours
RMSE	1.191849	0.901704	0.589311	0.349885
Mean	1.169402	0.884324	0.505794	0.337527

**Table 4:** The absolute trajectory error of various algorithms in Scenario 2.

Category	VINS-Mono	PL-VIO	ORB-SLAM3	Ours
RMSE	0.737751	0.605052	0.518785	0.199050
Mean	0.698574	0.576665	0.490459	0.189343

## Data Availability

Data are contained within the article.
